# M1 macrophage recruitment correlates with worse outcome in SHH Medulloblastomas

**DOI:** 10.1186/s12885-018-4457-8

**Published:** 2018-05-08

**Authors:** Chanhee Lee, Joongyub Lee, Seung Ah Choi, Seung-Ki Kim, Kyu-Chang Wang, Sung-Hye Park, Se Hoon Kim, Ji Yeoun Lee, Ji Hoon Phi

**Affiliations:** 1 Division of Pediatric Neurosurgery, Seoul National University Children’s Hospital, 101 Daehakro, Jongno-gu, 110-744 Seoul, Republic of Korea; 2 Medical Research Collaborating Center, Seoul National University Hospital, Seoul, South Korea; 3 Department of Pathology, Seoul National University College of Medicine, Seoul, Republic of Korea; 4 Department of Pathology, Yonsei University, College of Medicine, Severance Hospital, Seoul, Republic of Korea; 5 Department of Anatomy, Seoul National University College of Medicine, Seoul, South Korea

**Keywords:** Medulloblastoma, Sonic hedgehog, Macrophage, Recruitment, Prognosis

## Abstract

**Background:**

Recent progress in molecular analysis has advanced the understanding of medulloblastoma (MB) and is anticipated to facilitate management of the disease. MB is composed of 4 molecular subgroups: WNT, SHH, Group 3, and Group 4. Macrophages play a crucial role in the tumor microenvironment; however, the functional role of their activated phenotype (M1/M2) remains controversial. Herein, we investigate the correlation between tumor-associated macrophage (TAM) recruitment within the MB subgroups and prognosis.

**Methods:**

Molecular subgrouping was performed by a nanoString-based RNA assay on retrieved snap-frozen tissue samples. Immunohistochemistry (IHC) and immunofluorescence (IF) assays were performed on subgroup identified samples, and the number of polarized macrophages was quantified from IHC. Survival analyses were conducted on collected clinical data and quantified macrophage data.

**Results:**

TAM (M1/M2) recruitment in SHH MB was significantly higher compared to that in other subgroups. A Kaplan-Meier survival curve and multivariate Cox regression demonstrated that high M1 expressers showed worse overall survival (OS) and progression-free survival (PFS) than low M1 expressers in SHH MB, with relative risk (RR) values of 11.918 and 6.022, respectively.

**Conclusion:**

M1 rather than M2 correlates more strongly with worse outcome in SHH medulloblastoma.

**Electronic supplementary material:**

The online version of this article (10.1186/s12885-018-4457-8) contains supplementary material, which is available to authorized users.

## Background

Medulloblastoma (MB) is the most common pediatric brain malignancy that frequently arises below 10 years of age [1, 2]. Approximately 20–30% of patients remain incurable, and high dose radiation and chemotherapy frequently lead to significant long-term sequelae [3]. Progress in molecular diagnostics has revealed that MB is classified into 4 subgroups: WNT, SHH, Group 3 (G3) and Group 4 (G4) [1, 2, 4]. The prognosis of each subgroup ranges from being excellent in WNT MB to intermediate in SHH and G4, to poor in G3 MB [1, 4]. As subgroup-specific prognostication and personalized medicine are in demand, clinically applicable subgrouping has become essential [3, 5–7]. Practical molecular subgrouping has been developed by multiple researchers via screening subgroup-specific signature genes using various tools, such as nanoString nCounter [3, 4, 6].

The significance of lymphocytes and tumor-associated macrophages (TAMs) in the tumor microenvironment has been perpetually examined for more than a decade; however, their comprehensive role is rather elusive [8–12]. TAMs release growth factors, cytokines, and inflammatory mediators into the environment and are classified according to their functional phenotype [13–16]. The current paradigm of macrophage polarization is undergoing reassessment. It has been commonly accepted that classically activated M1 macrophages suppress tumor growth and progression by production of reactive oxygen species (e.g., nitric oxide), whereas alternatively activated M2 macrophages promote tumor growth and progression by releasing growth factors (e.g., epidermal growth factor, fibroblast growth factor 1, vascular endothelial growth factor A) [9, 13–16]. The literature has often described conflicting roles of TAMs in various cancers due to the complexity of the tumor microenvironment and diverse contributing factors, such as immune responses, tumor stages, and types of tumors [11, 13, 17–20].

Despite the molecular insights provided by MB subgroups, relatively little is known about the role of tumor microenvironment with respect to MB and its subgroups [8]. A previous report on the characterization of immunophenotype in pediatric brain tumors suggests that MB is less infiltrated with T lymphocytes and displays an immunosuppressive M2 phenotype compared to other pediatric brain tumors [8]. A recent study demonstrated that TAM recruitment is subgroup-specific in MB, suggesting that the expression of TAM-associated genes was significantly higher in the SHH subgroup [3]. This finding indicates that SHH MB has a distinct tumor microenvironment, which may have important pathophysiological and therapeutic implications. However, the roles of TAMs and their activation phenotypes are inconclusive because the previous study did not present the prognostic connotations of TAMs in SHH MB [3].

In the present study, we investigate the correlation between TAM recruitment in SHH MB with prognosis. We identified that M1 macrophage recruitment rather than total TAM recruitment correlates more strongly with a reduced overall survival outcome within the SHH subgroup. Considering the commonly accepted role of macrophage polarization in various human cancers (M1 tumor-suppressing and M2 tumor-promoting roles), the negative prognostic implication of M1 macrophages in SHH MB is intriguing and requires further investigation.

## Methods

### Patients and samples

The Institutional Review Board (IRB) of the Seoul National University Hospital (SNUH) approved the study protocol (IRB approval No. 1610–027-797). To identify SHH MB, 48 snap-frozen MB tissues were retrieved from the Brain Bank of the Department of Neurosurgery, Seoul National University Hospital. Tissue samples were collected from 141 MB patients who underwent surgery at Seoul National University Children’s Hospital (SNUCH) from 1999 to 2015. The molecular subgroups of the samples were partially verified via immunohistochemistry (IHC) using representative markers [4]. To solidify the molecular subgroup, a nanoString-based RNA assay was performed on these samples. Previously, we provided MB tissues to Dr. M. Taylor from the Hospital for Sick Children (Toronto, Canada) for analysis, and the molecular subgroups were provided for these cases through nanoString [10].

We collected 32 SHH MBs from two sources: cases newly tested for subgrouping (*n* = 16) and cases with subgroup information from Toronto (*n* = 16). Among the 32 known SHH MB patients, 25 patients had available formalin-fixed paraffin-embedded (FFPE) tissues. Two FFPE tissue samples were removed from selection due to small tissue size or the inability to undergo a complete experiment; 23 SHH MB samples were finally recruited from our institution. An additional 7 SHH MB FFPE tissue samples were received from Yonsei University. In total, 30 SHH MB were analyzed in the present study. Subgroups other than SHH were randomly selected with respect to FFPE tissue availability as control groups to validate the correlation between TAM infiltration and the prognosis of the subgroups (WNT = 3, Group 3 = 2, Group 4 = 17).

### Subgrouping

Molecular subgroups were identified through gene profiling using nanoString nCounter [6]. Total RNA was extracted from snap-frozen patient tissue samples (*n* = 48) using the miRNeasy kit according to the manufacturer’s protocol (Life Technologies, Carlsbad, CA, USA). Procedures related to hybridization, detection and scanning were performed as recommended by nanoString Technologies (Seattle, WA, USA). The collected data were normalized in *R*, and an algorithm for class prediction analysis was provided by Dr. M. Taylor (Toronto, Canada) [6]. The subgroup of additionally received FFPE tissue samples from Yonsei University, which were identified via immunohistochemistry (IHC), was provided by Dr. SH Kim (Seoul, Korea). For the SHH subgroup, IHC generally yields stable and concordant results with nanoString.

### Immunohistochemistry

Macrophage recruitment was investigated using immunohistochemistry (IHC) on FFPE tissue samples (*n* = 45). Human tonsil tissue was used as a positive control (Additional file 1: Figure S1).The recruitment of activated macrophages was identified using the following antibodies: CD68 for total macrophages, CD86 for M1-activation, and CD163 for M2-activation (Additional file 2: Table S1). Five hot spots were randomly selected in each paraffin section, and positive cells among the 300 counterstained cells were counted using the ImageJ Cell Counter plugin [21]. The mean value of the five hot-spots count was used in the following statistical analyses. Researchers engaged in the present experiment were blinded from all clinical data, including subgroup, through data collection.

### Immunofluorescence

To confirm the independent localization of M1 and M2 macrophages, an immunofluorescence (IF) assay was performed on FFPE tissue samples. The retrieved blocks were sectioned at 4 μm using a microtome and transferred to silane-coated slides by the SNUH pathology lab. The slides were deparaffinized in xylene and rehydrated through a graded ethanol series. To retrieve antigen, the slides were microwaved in 10 mM sodium citrate buffer (pH 6.0) for 3 min, with a 15 s cooling interval after 2 min. The slides were washed three times in phosphate-buffered saline (PBS) with 0.1% bovine serum albumin (BSA) for 5 min each and then permeabilized (1× PBS/ Timerasol: 95 mg/L, saponin: 0.6 g/L, normal goat serum: 1%) for 15 min. The slides were subsequently blocked in blocking solution (1 × PBS/ Timerasol: 95 mg/L, saponin: 0.35 g/L, normal goat serum: 3.5%) for 30 min at room temperature [22]. The primary antibody was prepared in a modified blocking solution (1 × PBS/ Timerasol: 95 mg/L, saponin: 0.1 g/L, normal goat serum: 1%), with adequate dilution and incubated overnight at 4 °C. The secondary antibody was similarly diluted accordingly and applied for 1 h at room temperature.

### Clinical data

Clinical data, including sex, age at diagnosis, pathology, degree of surgical resection, presence of leptomeningeal seeding at presentation, applied treatment modalities, progression, and survival, were collected independently of the researchers conducting the experiments. Progression-free survival (PFS) refers to the time interval from the day of initial surgery to the date when tumor progression was radiologically identified or the date of the last follow-up [10]. Overall survival (OS) refers to the time interval from the day of initial surgery to the date of patient death or the date of the last follow-up [10].

All 32 patients with SHH MB received chemotherapy. The chemotherapy regimens changed from 1999 to 2015. Prior to 2006, the Children’s Cancer Group (CCG) 9921 regimen (3 patients) or the 8 in 1 (6 patients) regimen were applied, and from 2006, the KSPNO (Korean Society for Pediatric Neuro-Oncology) protocols for infant or child MB were applied (14 patients). Eleven patients were aged < 3 yrs. at diagnosis, and radiation therapy (RT) was delayed for these patients. Overall, 20 patients received RT. The RT doses were adapted to the risk status of each patient: the standard risk group: craniospinal axis 19.8–23.4 Gy, tumor bed boost up to 54 Gy; the high risk group: craniospinal axis 28.8–36 Gy, tumor bed boost up to 54 Gy. The three patients for whom RT was delayed did not receive RT. One patient was lost to follow-up prior to initiating RT, while another patient died at 11 months with rapid disease progression, and another patient was cured with chemotherapy alone.

### Statistical analysis

Subgroup prediction analysis was conducted in *R*. IBM SPSS Statistics version 23 was used to perform common statistical analyses, including χ^2^, bivariate Pearson’s correlation, Cox regression analysis, survival analysis, and the log-rank test as previously described [10]. Appropriate indications are provided in the text and supplementary data.

## Results

### Identification of molecular subgroupsusing nanoString nCounter

To identify SHH MB, we performed gene profiling on 22 subgroup-specific signature genes on selected samples (*n* = 48) using nanoString nCounter [6]. We identified 5 WNT, 16 SHH, 5 Group3, and 26 Group4 MBs through class prediction analysis (Additional file 1: Figure S2); 7 of the 16 patients identified as SHH subgroup had adequate FFPE tissue samples available. Additionally, 16 SHH samples previously identified by the same method in Toronto were incorporated in the present study, yielding a total number of 23 SHH samples. Moreover, 22 randomly selected non-SHH subgroup samples were analyzed as control groups for the reliability and validity of IHC/IF techniques and counting. The subgroup of the validation cohort was pre-identified by immunohistochemistry only.

### Activated macrophage recruitment in the medulloblastoma subgroups

**Fig. 1:**
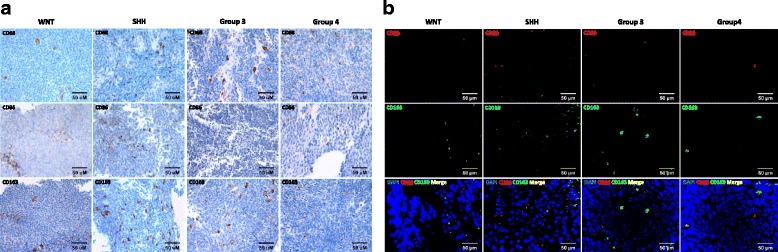
TAM recruitment across MB subgroups. (**a**) Representative CD68, CD86, and CD163 IHC images in WNT (*n* = 3), SHH (*n* = 23), Group 3 (*n* = 2) and Group 4 (*n* = 17) subgroups. Scale bar, 50 μm (**b**) Representative CD86 and CD163 IF images in each subgroup. Scale bar, 50 μm

**Fig. 2:**
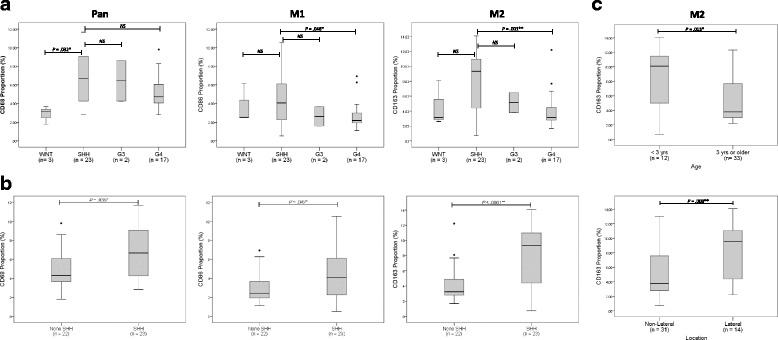
Proportion of TAM recruitment in MB. (**a**) Comparison of macrophage recruitment between the 4 subgroups. The one-way ANOVA results are presented; however, due to the small number of WNT and Group 3 subgroup samples, statistical significance is neglected. (**b**) Comparison between SHH and non-SHH subgroups; CD68 (*P* = .035), CD86 (*P* = .042), and CD163 (*P* < .0001) were significantly higher in the SHH subgroup than those in the non-SHH subgroups. (**c**) CD163-positive macrophages were significantly higher in patients younger than 3 years-of-age (*P* = .015) as well as the lateral location of the tumor (*P* = .008)

First, we investigated the unique recruitment pattern of tumor-associated macrophages (TAM) in the different MB subgroups. Immunohistochemistry (IHC) analysis was conducted to identify macrophage recruitment (Fig. 1a), and the recruited proportion of CD68-, CD86-, and CD163-positive macrophages were quantified in each subgroup (Fig. 2a & b). The comparison was largely SHH to G4 since the numbers of other subgroups was limited. Notably, CD163-positive M2 macrophages were significantly higher in the SHH subgroup (*n* = 23) compared to that in another subgroup (*n* = 22) (*P* < .001)*.* M1 macrophage recruitment was also significantly higher in the SHH subgroup than that in non-SHH subgroups (*P* = .048). Through immunofluorescence (IF) analysis, we confirmed that M1 and M2 macrophages, identified by CD86 and CD163, respectively, were located in different areas and were independently distinguishable (Fig. 1b).

### TAM recruitment and patient characteristics

The M2 macrophage proportion also correlated with patients < 3 years of age (*P* = .015) and the lateral location of the tumor (*P* = .008), which are known indicators of the SHH subgroup (Fig. 2c). We verified that the present quantification method and results were consistent with the findings of a previous study that used a different quantification method [3].

### TAM recruitment and survival outcomes in MB

Statistical analysis was conducted on the collected data to demonstrate the correlation between TAM in MB and prognosis. We dichotomously defined patient groups of high and low macrophage expressers based on the median-value of the macrophage counts. OS and PFS analyses on counted M1 and M2 activation markers were performed using the Kaplan-Meier plot and log-rank test (Additional file 1: Figure S3). MB patients with high M1 counts showed a considerable trend with shorter OS (*P* = .064). However, patients with high M2 counts showed shorter PFS (*P =* .037), which did not affect the OS of these patients. Considering that approximately half of all included cases were of the SHH subgroup and TAM is overrepresented only in this subgroup, the prognostic implications may be more clear in the SHH subgroup.

### TAM recruitment and survival outcomes in SHH MB

**Fig. 3:**
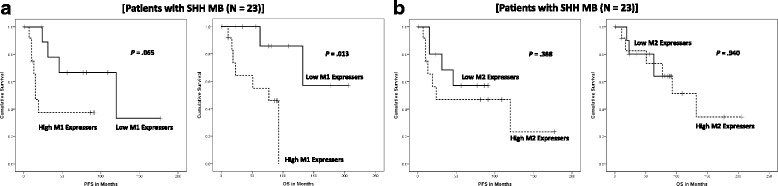
TAM recruitment and prognostic outcomes in SHH MB. (**a**) PFS and OS analyses using Kaplan-Meier plots and the log-rank test based on the CD86-positive macrophage counts. High M1 recruitment is correlated with shorter PFS (*P* = .065) and OS (*P* = .013). (**b**) PFS and OS analyses based on the CD163-positive macrophage counts. M2 recruitment did not show an obvious correlation with the prognostic outcome

We investigated whether SHH-specific macrophage recruitment showed a correlation with the survival outcomes in SHH MB (Fig. 3). High M1 expressers had shorter OS (*P* = .013) and a trend with shorter PFS (*P* = .065). Prognostic factors those are known to affect the outcomes, such as sex, age, and leptomeningeal seeding, were incorporated in multivariate Cox regression analysis (Tables 1 & 2). Interestingly, high M1 macrophages were significantly correlated with shorter OS (*P* = .030, RR = 11.918, 95% CI = 1.265–112.282) and PFS (*P =* .027, RR = 6.022, 95% CI = 1.232–29.433) in the SHH subgroup (Fig. 4). M2 macrophage recruitment did not show an obvious correlation with the outcome of SHH subgroup patients (Fig. 3b, Table 2).

**Table 1:** Relative risks for shorter PFS in SHH MB estimated with a Cox proportional hazards model

Clinical Factor	Univariate analysis				Multivariate analysis		
	*P* value	RR	95% CI		*P* value	RR	95% CI
M1 Count	.085	3.331	.848–13.091		.027	6.022	1.232–29.433
M2 Count	.399	1.727	.485–6.151		.246	2.361	.553–10.076
Age	.700	1.029	.890–1.190		.687	1.031	.890–1.194
Sex (Male)^a^	.098	.316	.081–1.237		.117	.256	.046–1.408
Leptomeningeal Seeding	.319	1.994	.514–7.736		.521	1.721	.328–9.037

*RR* Relative risk, *CI* Confidence interval

^a^Sex was included in the multivariate analysis model as a basic variable

**Table 2:** Relative risks for shorter OS in SHH MB estimated with a Cox proportional hazards model

Clinical Factor	Univariate analysis				Multivariate analysis		
	*P* value	RR	95% CI		*P* value	RR	95% CI
M1 Count	.038	9.558	1.128–81.002		.030	11.918	1.265–112.282
M2 Count	.940	1.060	.234–4.801		.601	.620	.104–3.710
Age	.111	1.146	.969–1.354		.180	1.118	.950–1.316
Sex (Male)^a^	.044	.117	.014–0.948		.080	.110	.009–1.298
Leptomeningeal Seeding	.064	4.693	.917–24.026		.422	2.097	.344–12.786

*RR*, Relative risk; *CI*, Confidence interval

^a^Sex was included in the multivariate analysis model as a basic variable

### TAM recruitment and other prognostic factors in SHH MB

With respect to the TAM infiltration within MB, other prognostic factors were investigated to identify potential correlations (Table 3). A multivariate analysis using binary logistic regression revealed that age, lateral tumor location, and large residual tumor (> 1.5cm^2^) were not significantly related to the M1 and M2 macrophage recruitment patterns in SHH MB (Additional file 2: Table. S2).

**Table 3:** Patient characteristics according to activated macrophage recruitment in SHH

Macrophage Polarization	CD86 (M1 macrophages)			CD163 (M2 macrophages)	
	High Expressers^a^	Low Expressers^b^		High Expressers^a^	Low Expressers^b^
Number	12	11		12	11
Mean	6.4 ± .6	2.1 ± .3		11.2 ± .4	4.6 ± .7
Age	5.0 ± 1.1	4.6 ± 1.5		5.7 ± 1.4	4.0 ± 1.2
M:F	7:5	5:6		7:5	5:6
Lateral tumor location	7 (30%)	7 (30%)		8 (35%)	6 (26%)
Gross total resection	10 (43%)	6 (26%)		9 (39%)	7 (30%)
Large residual tumor (> 1.5cm^2^)	1 (4%)	3 (13%)		2 (9%)	2 (9%)
Leptomeningeal Seeding	3 (13%)	2 (9%)		3 (13%)	2 (9%)

^a^High expressers indicates patients with greater than or equal to median count.

^b^Low expressers indicates patients with lower than median count

### TAM and survival outcome correlation in other cohort

To further confirm the correlation between TAM recruitment and survival outcome, 7 additional SHH MB from other cohorts were separately investigated (Additional file 1: Figure S4). Due to the short follow-up (FU) period and small population, the correlation between TAM recruitment and survival outcome was not significant, but the survival graphs showed a considerable trend with the current study cohort.

## Discussion

We demonstrate an unconventional correlation between subgroup-specific recruitment of TAM in SHH MB and prognosis. We confirmed subgroup-specific augmentation of M1 and M2 macrophages in SHH MB and compared this result with relevant prognostic factors. Survival analyses and Cox-regression analysis showed that M1 rather than M2 infiltration correlates better with worse OS and PFS in SHH MB, with relative risk values of 11.918 and 6.022, respectively.

The SHH MB subgroup, as suggested by its name, is thought to be driven by alterations in the Sonic-hedgehog signaling pathway [4]. The SHH pathway plays a crucial role in cerebellar development, inducing the proliferation of neuronal precursors [1, 4]. Individuals with germline or somatic mutations in the SHH pathway, such as *PTCH*, *SMO*, *SUFU*, *GLI1*, and *GLI2*, are predisposed to MB [1, 4]. Moreover, SHH MB has an intermediate prognosis among the 4 subgroups but, interestingly, is saturated with the highest number of TAMs, as demonstrated in the present and the previous one [1, 3]. A dichotomous age distribution (< 4 years and > 16 years) is another hallmark of SHH subgroup; the present study showed that the age distribution within SHH MB did not significantly correlate with activated macrophage recruitment [1].

The recognition of microenvironment in tumor biology has escalated over the past few decades, and this emphasis has led researchers to characterize contributing factors, including immunophenotypes, in various cancers [8]. However, these studies are often limited to phenotypic characterization and lacked prognostic connotation. A previous study investigated TAM recruitment in MB and proposed subgroup-specific recruitment in SHH MB [3]. We sought to verify this phenomenal recruitment in MB by a different method. Indeed, we found corroborating results showing augmented TAM recruitment in SHH MB and confirmed its unique microenvironment. Aside from M2 macrophages, we further characterized M1 macrophages in SHH MB and investigated the prognostic connotation of their recruitment.

In the present study, high M1 macrophages correlated with poor prognosis in SHH MB patients. This result apparently contradicts the common view of tumoricidal M1 macrophages. In many cancer types, M1 macrophage infiltration is associated with better prognosis [23–25]. However, recent studies suggest that the dichotomous M1/M2 classification is oversimplified, and the role of TAM in tumors is still controversial [14, 26]. We cannot provide a conclusive role for M1 macrophages in SHH MB because the causality of the worse prognosis associated with M1 macrophages has not been investigated. However, few plausible hypotheses can be made from the present results: 1) high M1 macrophage recruitment assists growth and progression of SHH MB contrary to its role in other cancers, 2) M1 macrophages are highly recruited to enhance the tumoricidal effect in aggressive group of SHH MB, but this mechanism alone was insufficient to fight the particular malignancy, or 3) high M1 recruitment is an epiphenomenon, and these cells are simply recruited by other SHH MB initiators and do not directly affect prognosis. Interestingly, the literature suggests multiple perspectives. The loss of nitric oxide synthase2 (NOS2) in the Ptch1^+/-^SHH MB mouse model was reported to promote development of medulloblastoma [27]. NOS2 is a key enzyme that produces nitric oxide in M1 macrophages in response to pathogens [26]. This suggests good prognostic role of M1 macrophages, which supports the second hypothesis. However, direct production of interferon-γ, a known stimulatory cytokine of M1 macrophages, in the developing brain was reported to activate the SHH pathway and cerebellar dysplasia. [28]. This activation may suggest that M1 macrophages are coincidentally recruited in response to the abnormal source of IFN-γ in the developing brain, not in recognition of MB or to destroy it. Such conflicting perspectives may also suggest a context-dependent role for TAM.

The small number of patients is a major limitation of the present study. The heterogeneity of the treatment administered to the patients may also confound the results, although all patients followed modernized treatment protocols in terms of risk stratification, chemotherapy regimen, and RT doses. Further validation in a comparable MB cohort is required to consolidate the role of TAM in SHH MB.

## Conclusion

High M1 macrophage recruitment correlated with a worse prognostic outcome in SHH MB. The present results are unconventional, yet intriguing, as the commonly accepted role of M1 macrophages should demonstrate the opposite effect. However, additional follow-up studies are required; the present study is limited because of its small sample size and strong dependence on the IHC results. Further in vitro and in vivo studies should be performed to determine the mechanism and causality of the worse prognostic outcome associated with M1 macrophages in SHH MB.

## Additional files

Additional file 1: **Figure S1.** Macrophage recruitment in human tonsil FFPE tissue. **Figure S2.** Expression heatmap of 22 subgroup-specific signature genes in 48 study patients by the nanoString nCounter System. **Figure S3.** TAM recruitment and prognostic outcomes in the whole patient cohort. **Figure S4.** TAM recruitment and prognostic outcomes in SHH MB from Yonsei University. (PPTX 3883 kb)

Additional file 2: **Table S1.** List of antibodies used for immunohistochemistry and immunofluorescence assay **Table S2.** Correlation between TAM and other prognostic factors estimated with a logistic regression in SHH MB. (DOCX 17 kb)

## Abbreviations

CD163: Cluster of differentiation 163
CD68: Cluster of differentiation 86
CD86: Cluster of differentiation 86
FFPE: Formalin-fixed paraffin-embedded
G3: Group 3
G4: Group4
IF: Immunofluorescence
IHC: Immunohistochemistry
IRB: Institutional review board
MB: Medulloblastoma
NOS2: Nitric oxide synthase2
OS: Overall survival
PBS: Phosphate-buffered saline
PFS: Progression-free survival
RR: Relative risk
SHH: Sonic hedgehog
TAM: Tumor-associated macrophages
WNT: Wingless/ Integrated
