# Supplementary material for: M1 macrophage recruitment correlates with worse outcome in SHH Medulloblastomas
Source: BMC Cancer. 2018 May 8;18:535. doi: 10.1186/s12885-018-4457-8 (PMC5941618; doi:10.1186/s12885-018-4457-8)
Supplement: Supplementary file 1 — Figure S1. Macrophage recruitment in human tonsil FFPE tissue. Figure S2. Expression heatmap of 22 subgroup-specific signature genes in 48 study patients by the nanoString nCounter System. Figure S3. TAM recruitment and prognostic outcomes in the whole patient cohort. Figure S4. TAM recruitment and prognostic outcomes in SHH MB from Yonsei University. (PPTX 3883 kb) [file 12885_2018_4457_MOESM1_ESM.pptx]

## Slide 1
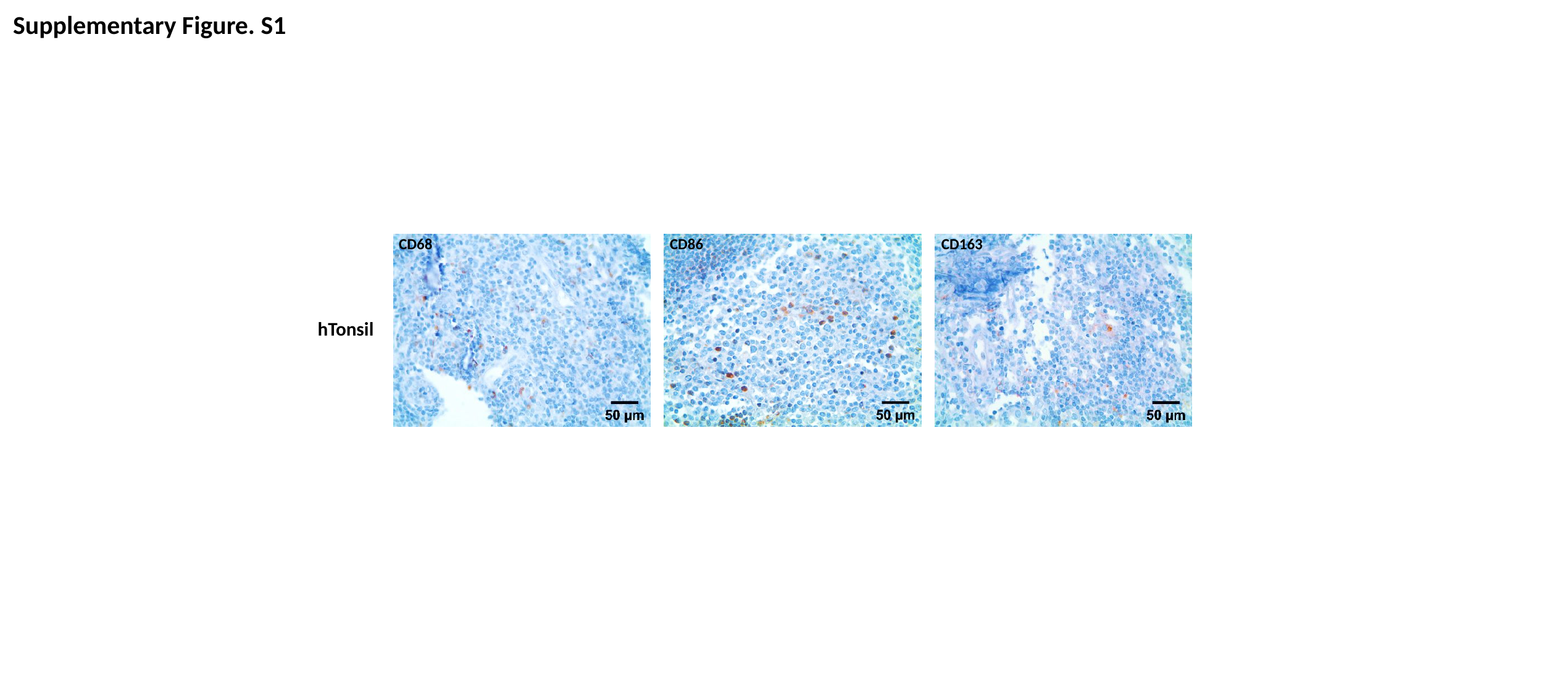

Supplementary Figure. S1
CD68
CD86
CD163
hTonsil

## Slide 2
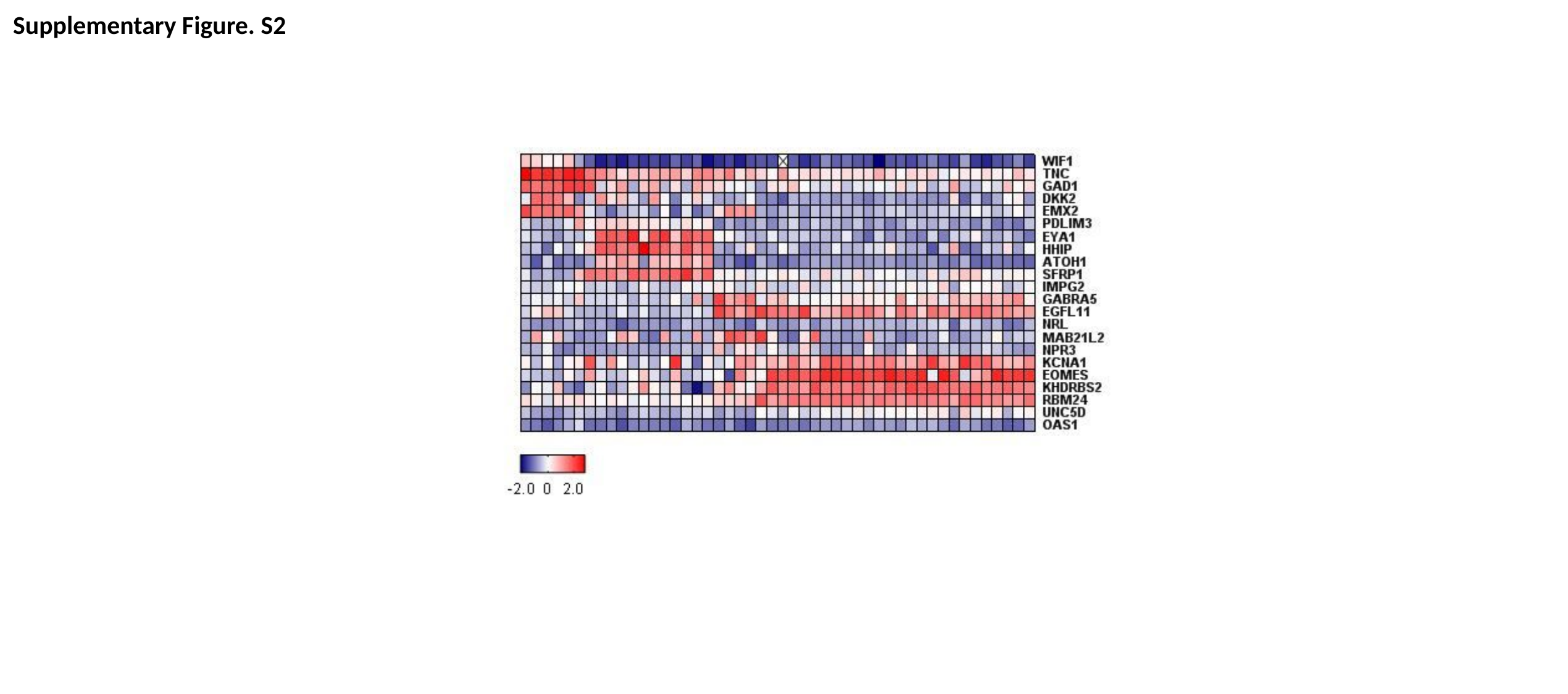

Supplementary Figure. S2

## Slide 3
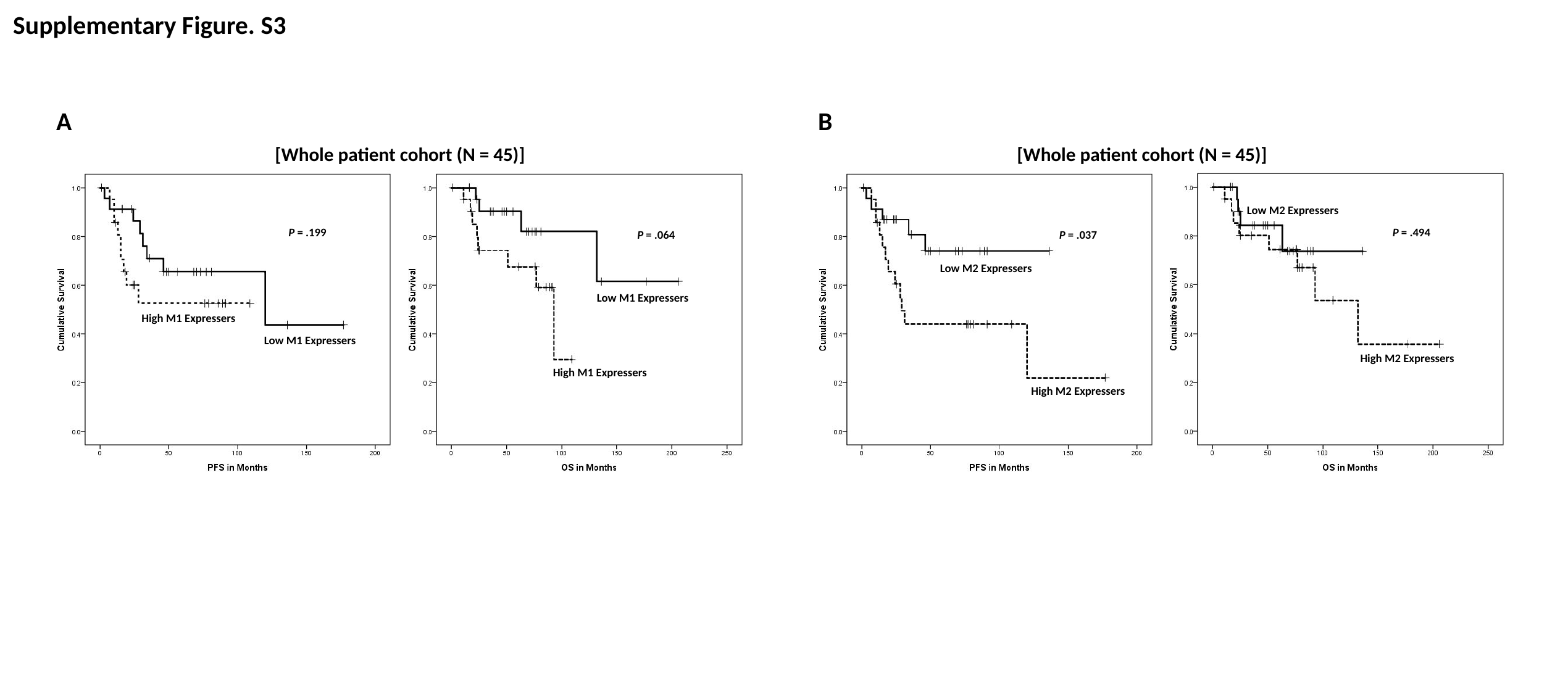

Supplementary Figure. S3
A
B
[Whole patient cohort (N = 45)]
[Whole patient cohort (N = 45)]
Low M2 Expressers
P = .199
P = .494
P = .064
P = .037
Low M2 Expressers
Low M1 Expressers
High M1 Expressers
Low M1 Expressers
High M2 Expressers
High M1 Expressers
High M2 Expressers

## Slide 4
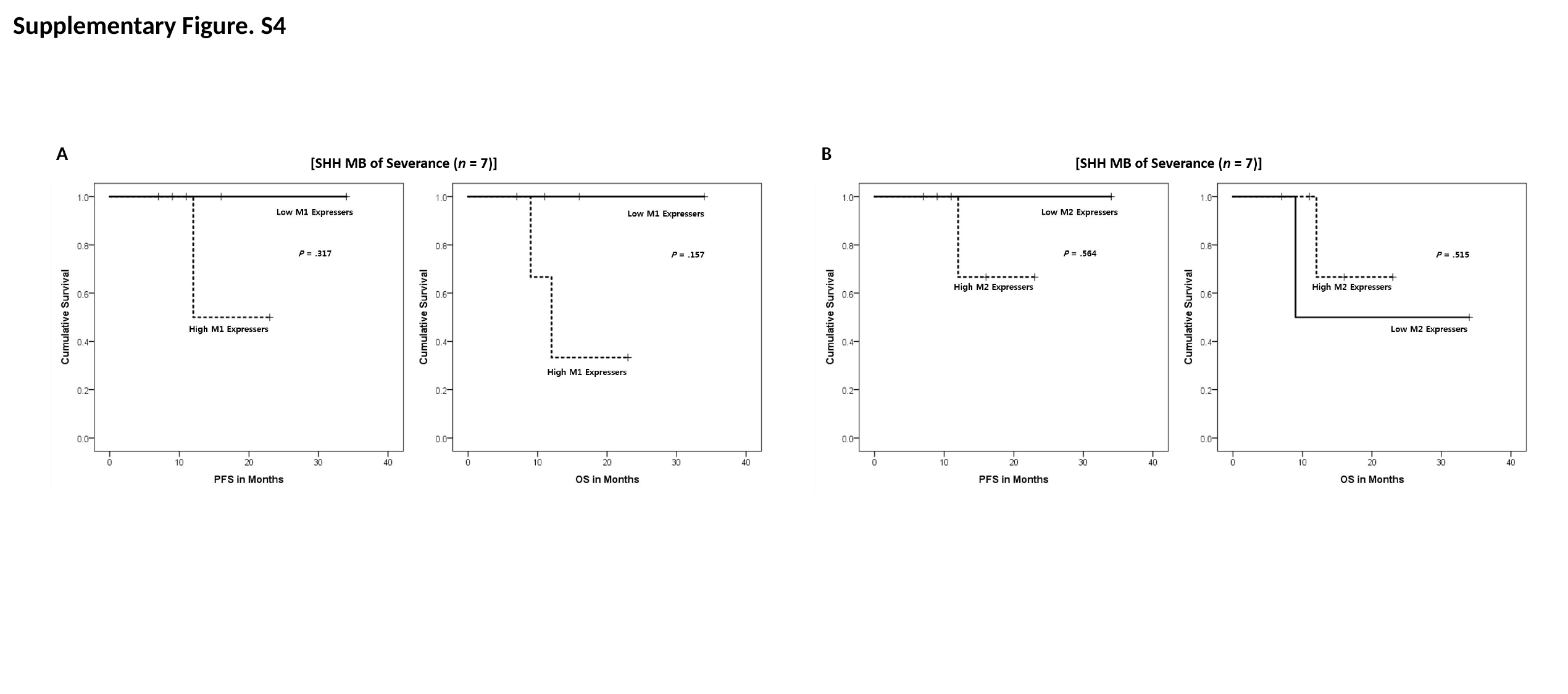

Supplementary Figure. S4
