# Supplementary material for: M1 macrophage recruitment correlates with worse outcome in SHH Medulloblastomas
Source: BMC Cancer. 2018 May 8;18:535. doi: 10.1186/s12885-018-4457-8 (PMC5941618; doi:10.1186/s12885-018-4457-8)
Supplement: Supplementary file 2 — Table S1. List of antibodies used for immunohistochemistry and immunofluorescence assay Table S2. Correlation between TAM and other prognostic factors estimated with a logistic regression in SHH MB. (DOCX 17 kb) [file 12885_2018_4457_MOESM2_ESM.docx]

**Suppl. Table S1.** List of antibodies used for immunohistochemistry and immunofluorescence assay

| **Antibodies** | **Supplier** | **Species** | **Dilution (IHC)** | **Dilution (IF)** | **2° Ab** | **Reference** |
| --- | --- | --- | --- | --- | --- | --- |
| CD68 | Abcam (San Diego, Ca) | Mouse | 1:200 | 1:400 | 1:5000 | ab955 |
| CD86 | LSBio (Seattle, WA) | Rabbit | 1:6000 | 1:1600 | 1:5000 | LS-B11911 |
| CD163 | Abcam (San Diego, Ca) | Mouse | 1:600 | 1:800 | 1:5000 | ab156769 |

**Suppl. Table S2.** Correlation between TAM and other prognostic factors estimated with a logistic regression in SHH MB

| **Macrophage Polarization** | **CD86 (M1 macrophages)** | | |  | **CD163 (M2 macrophages)** | | |
| --- | --- | --- | --- | --- | --- | --- | --- |
|  | ***P* value** | **RR** | **95% CI** |  | ***P* value** | **RR** | **95% CI** |
| Age | .775 | 1.034 | .823–1.298 |  | .490 | .926 | .744–1.152 |
| Sex (Male) ^a^ | .297 | 3.151 | .415–23.924 |  | .717 | 1.409 | .221–8.980 |
| Leptomeningeal Seeding | .353 | 3.044 | .291–31.845 |  | .731 | .680 | .076–6.102 |
| Gross total resection | .345 | 4.125 | .218–78.119 |  | .731 | 1.639 | .098–27.297 |
| Large residual tumor (>1.5cm^2^) | .763 | .540 | .010–29.432 |  | .979 | .953 | .026–34.275 |
| RR, relative ratio; CI, confidence interval.  ^a^Sex was included in the multivariate analysis model as a basic variable | | | | | | | |
